# Proximal and distal right bundle branch pacing: Insights into conduction system physiology

**DOI:** 10.1016/j.hrcr.2023.03.005

**Published:** 2023-03-14

**Authors:** Haran Burri, Nikola Kozhuharov, Marek Jastrzebski

**Affiliations:** ∗ Cardiac Pacing Unit, University Hospital of Geneva, Geneva, Switzerland; † First Department of Cardiology and Electrocardiology, Jagiellonian University, Krakow, Poland

**Keywords:** Conduction system pacing, Right bundle branch pacing, Left bundle branch area pacing, Right bundle branch block, Conduction system physiology

**Key Teaching Points**

• Conduction system pacing is possible from the proximal and from the distal right bundle branch (the latter is, however, rare).
• A QR pattern in lead V_1_, with a short R-wave peak time in V_6_ (indicating left-sided conduction tissue activation), is possible when pacing from the right-sided interventricular septum.
• This finding may possibly due to left-sided conduction fibers that penetrate the interventricular septum, or to early left-sided activation via a thin septum.
• Our case may open new perspectives for conduction system pacing in select patients who display these features.

## Introduction

Conduction system pacing (CSP) is progressively gaining mainstream practise for offering a more physiological form of antibradycardia pacing compared to right ventricular (RV) pacing, as well as an alternative to biventricular pacing for treating heart failure. CSP usually involves His bundle pacing or left bundle branch area pacing (LBBAP). Distal right bundle branch pacing (RBBP) has only been described to date in a case report.^1^ We describe herein a patient in whom proximal as well as distal RBBP was observed. Evidence of rapid left ventricular (LV) activation by pacing of the RV septum is also discussed.

## Case report

A 71-year-old patient with chronic atrial fibrillation with symptomatic brady-tachy syndrome was scheduled for single-chamber pacemaker implantation with LBBAP in anticipation of possible future atrioventricular nodal ablation. Baseline QRS morphology was normal with 93 ms duration. CSP procedures are all filmed at our institution with simultaneous recordings of fluoroscopy, electrophysiology bay tracings, pacing system analyzer screen, and video of the operation field, allowing precise reconstitution of the entire procedure.

**Figure 1:**
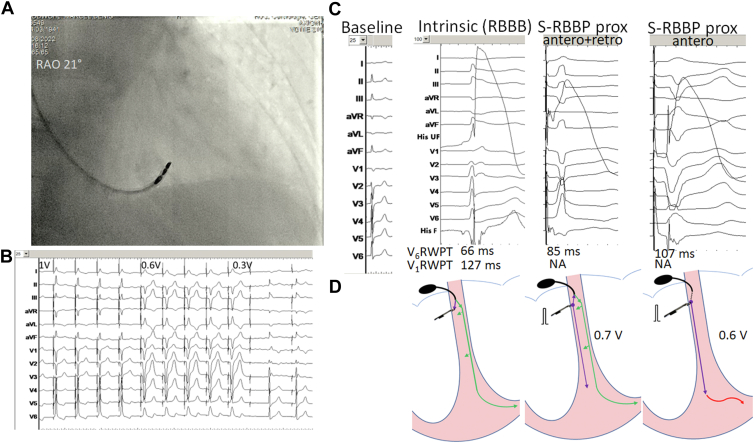
Findings with lead position at the proximal pacing site. **A:** Right anterior oblique (RAO) fluoroscopic view. **B:** Transition in paced QRS morphology with decrementing pacing output. Note traumatic right bundle branch block (RBBB) morphology of the intrinsic QRS observed after lead fixation. **C:** Electrocardiogram (ECG) morphology, endocardial signals, and R-wave peak time (measured from the potential in intrinsic rhythm or from the pacing spike). QRS is normal at baseline (ECG displayed at 25 mm/s). Following lead fixation, RBBB is observed (tracing displayed at 100 mm/s) with visualization of a right bundle branch potential (delay to QRS onset of 32 ms, with fragmentation probably resulting from injury). A relatively narrow QRS results from conduction tissue capture proximal and distal to the site of RBBB with anterograde (antero) and retrograde (retro) conduction. Capture is selective (S-RBBP)—note the split signal on the filtered conduction system pacing (CSP F) channel. At lower output (0.6 V), selective capture distally to the site of block results in left bundle branch block QRS morphology and wide splitting of the signal on the CSP F electrogram. **D:** Schematic representation of hypothetical activation with proximal RBBP. During intrinsic conduction, potential to QRS interval is short owing to simultaneous conduction down the right bundle (*purple arrow*) and the left bundle (*green arrows*) with early activation of the left septum. With RBBP and bidirectional propagation (proximal as well as distal to the conduction block), V_6_RWPT is longer than the potential to QRS interval owing to the sequential retrograde conduction via the right bundle and anterograde conduction down the left bundle. The ventricular potential on the CSP F channel is early owing to transseptal propagation. At low output with loss of capture proximal to the site of block, V_6_RWPT prolongs significantly owing to left ventricular activation occurring via myocardial propagation only (depicted by the *red arrow*). The signal in the CSP F channel splits significantly, as local myocardial activation now occurs by propagation from apex to base (rather than transseptally). Filter settings: CSP UF 0.5–300 Hz; CSP F 30–300 Hz.

After the tricuspid annulus had been identified by electrogram analysis (with presence of atrial and ventricular potentials), proximal LBBAP was initially attempted, using a Solia 60 cm lead and a Selectra 55 guiding catheter (Biotronik, Berlin, Germany). The lead was positioned below the annulus at about 10 mm toward the apex in the right anterior oblique view (Figure 1A). Traumatic right bundle branch block (RBBB) resulted from positioning the guiding catheter. After deploying of the helix, a fragmented potential of negative polarity of 21 ms duration, and with a potential to QRS delay of 32 ms, was visualized (Figure 1C). Pacing resulted in a narrow QRS, suggestive of HBP. Threshold testing showed a transition at 0.6 V / 0.5 ms from narrow QRS morphology to a left bundle branch block morphology, indicating RBBP distal to the site of block (Figure 1B).

**Figure 2:**
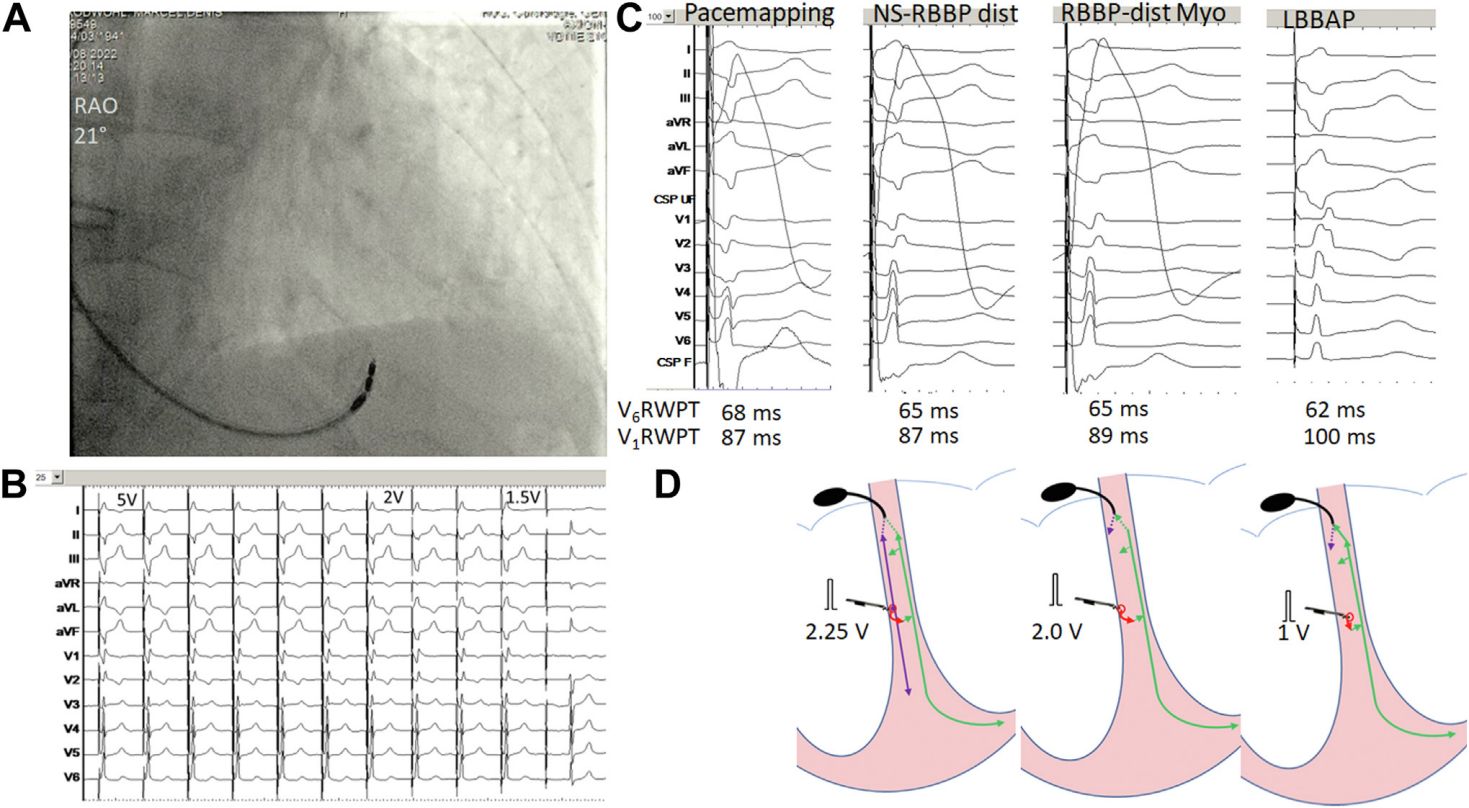
Findings with lead position at the distal pacing site. **A:** Lead position in the right anterior oblique (RAO) view. **B:** After screwing the lead superficially, slight transition in QRS morphology was observed at decrementing pacing output (visible in the inferior leads). Note recovery of traumatic right bundle branch block (RBBB) and resumption of baseline morphology of the intrinsic QRS. **C:** Electrocardiogram morphology, endocardial signals, and R-wave peak time (RWPT). First panel: QRS obtained with pace mapping of the right ventricular septum (before screwing the lead) with an unexpectedly short V_6_RWPT and a qr pattern in V_1_. Morphology with pace mapping was similar in the peripheral leads to that obtained after superficial lead insertion (second panel) and nonselective distal RBBP (NS-RBBP dist), with slight differences in the precordial leads (indicating earlier left ventricular activation after lead fixation within the septum). With decrementing output, myocardial capture only was obtained (RBBP-dist Myo, third panel). After additional rotations of the lead, left ventricular septal pacing (left bundle branch area pacing, LBBAP) was obtained (fourth panel), with change in QRS morphology and absence of QRS transitions with decrementing output. **D:** Schematic representation of hypothetical activation during pace mapping/distal RBBP and LBBAP. Left panel: During pace mapping and after superficial lead insertion at high output, RBBB and local myocardial capture are obtained, with transseptal retrograde activation of Purkinje fibers, resulting in left-to-right septal activation (explaining the QR/R pattern in V_1_ and short V_6_RWPT) but with a short V_6_–V_1_ interpeak interval owing to early right ventricular activation. At decrementing output after superficial lead insertion (middle panel), there is loss of right bundle branch capture. V_6_RWPTs are identical during NS-RBBP and Myo RBBP. With deeper lead insertion (right panel), distal RBBP is lost and LBBAP results in a slightly shorter V_6_RWPT owing to greater proximity of the lead to the Purkinje fibers, with prolongation of V_1_RWPT owing to delayed right ventricular activation. Filter settings as in Figure 1.

As it was considered that future atrioventricular nodal ablation could be possible and in order to avoid compromising lead function,^2^ a more distal lead position was sought. The lead was unscrewed and positioned 20 mm in an apical and inferior direction (Figure 2A). There was no presystolic potential visible at this site. Pace mapping at this spot (before screwing of the lead) unexpectedly revealed a relatively narrow QRS with a Qr pattern in V_1_ (Figure 2B). The lead was then fixated with a few rotations of the lead body. Paced QRS morphology in V_1_ showed a QR pattern, with an R-wave peak time (RWPT) in V_6_ of 65 ms (Figure 2B). Capture threshold was 1.2 V / 0.5 ms.

**Figure 3:**
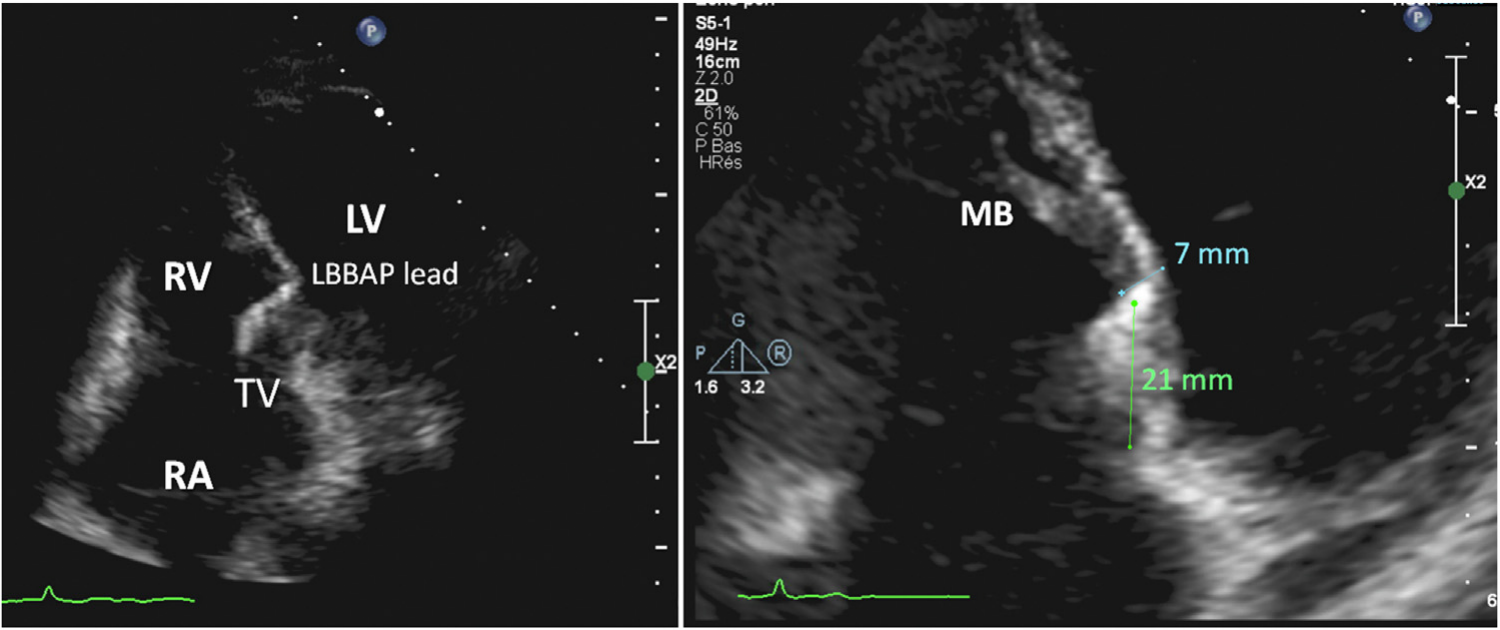
Left panel: Echocardiogram in the apical 4-chamber view showing left bundle branch area pacing (LBBAP) lead position. Right panel: Zoom showing lead tip in the left ventricular septal subendocardium, with insertion near the base of the moderator band (MB), 21 mm from the septal leaflet of the tricuspid valve (TV). Thickness of the interventricular septum was only 7 mm. LV = left ventricle; RA = right atrium; RV = right ventricle.

Upon retrospective review of the tracing, there had been a slight transition in QRS morphology in the inferior leads when the output had been reduced directly from 5 V to 2 V / 0.5 ms (Figure 2B and 2C), which was reproduced 2 more times during repeated testing but had initially gone unnoticed. Unipolar sensing from the anode did not show any current of injury, confirming superficial lead depth. Owing to a rise in threshold to 1.7 V / 0.5 ms, a few additional lead rotations were given, with an R pattern in V_1_ and V_6_RWPT shortening to 62 ms. Capture threshold rose to 2 V / 0.5 ms and then fell to 1.4 V / 0.5 ms over the next minutes, following which the lead was left in this position. A transthoracic echocardiogram was later performed and showed that the final lead position was at the left septal subendocardium, 21 mm from the insertion of the tricuspid septal leaflet. The thickness of the interventricular septum was only 7 mm at this site (Figure 3).

## Discussion

Based on analysis of lead position on fluoroscopy, intrinsic signals, paced QRS morphology at different outputs, and measurements of timing of RWPT in V_1_ and in V_6_ (as surrogates for anterior RV and lateral LV activation, respectively), the following deductions may be made:

(1) The initial lead position was on the proximal right bundle branch (RBB). The potential to QRS duration of 32 ms is in keeping with this hypothesis, which was later confirmed by selective RBBP with retrograde block and a typical left bundle branch block morphology at low output. This was owing to capture of conduction tissue distal to the site of traumatic RBBB. At higher output, the conduction tissue proximal to the block was also captured, narrowing the QRS.
(2) The lead had been repositioned on a more distal branch of the RBB, as indicated by the slight transition in QRS morphology with decrementing unipolar output, which is the hallmark of conduction tissue capture. Loss of RBB capture had, however, no impact on LV activation, as indicated by unchanged V_6_RWPT, which was surprisingly short.
(3) Left-sided conduction system capture seemed to be present at the site of the distal RBBP, already during pace mapping of the endocardial surface (before screwing in the lead).
(4) After a deeper lead position had been obtained, RBBP was lost, as indicated by lack of QRS transitions with decrementing output. The end result was LBBAP.

Our findings provide strong evidence for proximal and distal RBBP, as indicated by the transitions in QRS morphology with decrementing pacing output at the 2 sites. QRS morphology with proximal RBBP resembles that of HBP, but differs from the intrinsic QRS owing to the slightly modified ventricular activation sequence,^3^ and can be distinguished by a stimulus-V_6_RWPT that is significantly longer than the potential to V_6_RWPT (instead of being identical^3^; see Figure 1D for explanation).^4^ Distal RBBP from an RV septal lead has only recently been described in another case report.^1^ Owing to the very minor changes in QRS morphology with distal RBB capture compared to pure myocardial capture at the same pacing site (and no difference in the precordial leads), it seems unlikely that distal RBB capture has any meaningful clinical benefit.

Another novel observation was the Qr morphology in V_1_ and V_6_RWPT of 68 ms during pace mapping of the right-sided septal endocardium. We have previously found V_6_RWPT < 75 ms to be 100% specific for *left-sided* conduction system capture.^5^ The short V_6_RWPT could not have resulted from conduction by transseptal fibers from the RBB, as V_6_RWPT was long (107 ms) during selective proximal RBBP in the presence of RBBB with retrograde block (Figure 1C). Our hypothesis is that septal myocardial capture at this location was shortly followed by secondary retrograde activation of left-sided conduction tissue. Although intramural Purkinje fibers have never been identified in human hearts, either histologically^6^^,^^7^ or by micro–computed tomography,^8^ it is acknowledged that terminal fibers may be under-recognized by the former technique owing to its destructive nature, and by the latter by loss of fibrous sheaths.^8^ In the IMAGE-LBBP study evaluating LBBP lead depth by computed tomography, fascicular potentials were visualized when the lead tip was within the thickness of the septum, >4 mm from the LV blood pool, with QRS morphology compatible with LBBP, thus suggesting existence of penetrating conduction fibers.^9^

Another explanation could be that the relatively thin septum of our patient at the lead insertion site (7 mm) explained the findings. Durrer and colleagues^10^ have shown that septal activation usually proceeds from left to right, and that transseptal conduction velocity of stimulation impulses from right to left was on average 43.4 cm/s, with a conduction velocity of Purkinje fibers in the order of 2 m/s. Transseptal myocardial activation from the RV septum over 5–10 mm would reach the subendocardial left-sided Purkinje network within 10–20 ms. Activation of the left septal Purkinje network may then result in left-to-right septal activation, with a QR morphology in V_1_. Wavefront propagation to the LV free wall over approximately 10 cm of Purkinje network would require an additional 50 ms, resulting in a V_6_RWPT of ∼60–70 ms, which was within the range of our observations. However, it remains unexplained why RV septal pacing does not result in this ECG pattern more often. Nevertheless, it is of interest that a relatively physiological QRS may result from right-sided septal pacing, which may open a new avenue for CSP.
